# Prostate Tissue-Induced Platelet Activation and Platelet–Neutrophil Aggregation Following Transurethral Resection of the Prostate Surgery: An In Vitro Study

**DOI:** 10.3390/biomedicines13041006

**Published:** 2025-04-21

**Authors:** Po-An Lin, Hsiang-Han Huang, Mei-Hua Hu, Go-Shine Huang, En Meng, Yi-Lin Chiu, Yung-Chi Hsu, Wei-Hung Chan

**Affiliations:** 1 Department of Anesthesiology, Tri-Service General Hospital, National Defense Medical Center, Taipei 114202, Taiwan; poantoucheli@gmail.com (P.-A.L.); alan3413123@gmail.com (H.-H.H.); kshgodoc@gmail.com (G.-S.H.); x0939778570@gmail.com (Y.-C.H.); 2 Division of Pediatric General Medicine, Department of Pediatrics, Chang Gung Memorial Hospital at LinKou, College of Medicine, Chang Gung University, Taoyuan 33302, Taiwan; emerald@cgmh.org.tw; 3 Graduate Institute of Clinical Medical Sciences, College of Medicine, Chang Gung University, Taoyuan 33302, Taiwan; 4 School of Chinese Medicine, College of Medicine, Chang Gung University, Taoyuan 33302, Taiwan; 5 Division of Urology, Department of Surgery, Tri-Service General Hospital, National Defense Medical Center, Taipei 114202, Taiwan; mengen@mail.ndmctsgh.edu.tw; 6 Department and Graduate Institute of Biochemistry, National Defense Medical Center, Taipei 114202, Taiwan; 7 Department of Biochemistry, National Defense Medical Center, Taipei 114201, Taiwan; yilin1107@mail.ndmctsgh.edu.tw

**Keywords:** TURP, disseminated intravascular coagulation, platelet activation, P-selectin expression, platelet–leukocyte aggregation, platelet–neutrophil interaction

## Abstract

**Background**: This study aimed to investigate the effects of prostate tissue on platelet activation markers, primarily assessed through P-selectin expression, and to assess the formation of platelet–leukocyte aggregations in response to prostate tissue exposure. Furthermore, we compared platelet activation induced by prostate tissue homogenates with that induced by thrombin stimulation. These processes may play a role in the development of disseminated intravascular coagulation (DIC) following transurethral resection of the prostate (TURP). **Methods**: We collected prostate tissue samples from 12 patients undergoing TURP. The samples were homogenized and used to stimulate platelet-rich plasma in vitro. Flow cytometry was used to measure platelet P-selectin expression and platelet–leukocyte aggregation. Additionally, four experimental groups were established: (A) saline control, (B) thrombin stimulation, (C) phosphate-buffered saline (PBS) control, and (D) prostate tissue homogenate. Data were analyzed to assess the impact of prostate tissue and thrombin on platelet activation and platelet–leukocyte interactions. **Results**: Prostate tissue homogenates significantly increased platelet P-selectin expression and platelet–neutrophil aggregation compared with the control groups (*p* < 0.05). Overall, platelet–leukocyte aggregation was not significantly different between the thrombin and prostate tissue groups. However, prostate tissue exposure did not significantly affect platelet–monocyte and platelet–lymphocyte aggregations. **Conclusions**: Prostate tissue exposure during TURP induces platelet activation, particularly platelet P-selectin expression and platelet–neutrophil aggregation, suggesting a potential mechanism for DIC development. These findings highlight the importance of monitoring platelet activity in patients undergoing TURP and indicate that interventions targeting platelet P-selectin expression and platelet–neutrophil interactions may help mitigate DIC risk.

## 1. Introduction

Benign prostatic hyperplasia (BPH) is a common condition in aging men that often leads to bladder outlet obstruction. Transurethral resection of the prostate (TURP) remains the standard surgical intervention for severe cases of BPH-induced bladder outlet obstruction. Although effective, TURP is associated with several potential complications, including disseminated intravascular coagulation (DIC), urinary tract infections, sepsis, and significant bleeding [1,2,3,4,5,6]. DIC is a severe and life-threatening complication during or after TURP, with mechanisms that are not yet fully understood [4,5].

DIC is characterized by widespread intravascular coagulation, which is triggered by exposure to procoagulants that are insufficiently counterbalanced by natural anticoagulant mechanisms and endogenous fibrinolysis [7,8,9]. The pathology involves significant platelet activation and platelet count reduction, similar to thrombotic and inflammatory states in sepsis [10]. The cascade begins when the integrity of the endothelium is compromised, such as during TURP surgery, wherein endothelial cells can also be injured, therefore leading to coagulation pathway activation, with tissue factor playing a central role, eventually leading to thrombin generation, which amplifies coagulation and inflammation, primarily through platelet activation [7,8,9,11,12,13].

Platelets, as key players in both coagulation and inflammation, express P-selectin (CD62P) upon activation, a molecule critical for platelet–leukocyte aggregation [7,13,14,15]. These aggregations can occur between platelets and various leukocyte subpopulations, including neutrophils, monocytes, and lymphocytes. Platelet–leukocyte interactions, mediated primarily through platelet P-selectin binding to P-selectin glycoprotein ligand-1 (PSGL-1) on leukocytes, have been implicated in various clinical scenarios beyond TURP-associated DIC. For example, in sepsis, platelet–neutrophil aggregates contribute significantly to inflammatory responses, microvascular thrombosis, and organ dysfunction by enhancing neutrophil extracellular trap (NET) formation and pro-inflammatory cytokine release [13,14]. In cardiovascular diseases, particularly acute coronary syndromes, increased platelet–leukocyte aggregation correlates with heightened vascular inflammation, plaque instability, and adverse clinical outcomes, highlighting the pivotal role of these interactions in both thrombotic and inflammatory pathways [16,17]. This broader biological framework underscores the pivotal role of platelet–leukocyte interactions, potentially elucidating shared mechanisms of inflammatory-mediated coagulopathy in diverse clinical settings. Although TURP has been previously linked to DIC, the precise mechanisms through which prostate tissue or related factors, such as thrombin, contribute to platelet activation and subsequent DIC remain unclear [7,13,14,15].

In this study, we hypothesize that the prostate tissue contributes to increased platelet activation, evidenced by P-selectin expression and platelet–leukocyte aggregation. Moreover, this mechanism may involve thrombin. We aimed to investigate the effects of prostate tissue on platelet activation markers, primarily assessed through P-selectin expression, and the formation of platelet–leukocyte aggregations in response to prostate tissue exposure.

## 2. Materials and Methods

### 2.1. Reagents and Flow Cytometry

We used the anti-CD41a-PE or anti-CD41a-FITC antibody (Becton Dickinson [BD], Franklin Lakes, NJ, USA), a platelet-specific monoclonal antibody (mAb) that recognizes the platelet GPIIb/IIIa complex, independent of activation. To detect P-selectin, we used anti-CD62P-PE (BD), a monoclonal antibody targeting the surface-expressed P-selectin on activated platelets. We also used the anti-CD45-FITC (BD), a monoclonal antibody against the leukocyte common antigen, independent of leukocyte activation. Additionally, IgG1-FITC and IgG1-PE antibodies (BD) were utilized to control nonspecific binding. Subsequently, flow cytometry analysis was performed using a FACSCalibur flow cytometer (BD) equipped with a standard two-color filter configuration. Data were analyzed using BD CellQuest software (version 5.1). We used thrombin (Sigma, St. Louis, MO, USA) to stimulate platelets. Prostate tissue samples were homogenized using a homogenizer (PRO-PK-01200S, Pro Scientific, Oxford, CT, USA).

### 2.2. Preparation of Prostate Tissue Homogenates and Blood Samples

The Ethics Committee of Tri-Service General Hospital, Taipei, Taiwan, approved this study (TSGHIRB No: 2-103-05-041), which followed the approved guidelines. The study was registered on ClinicalTrials.gov (identifier: NCT05059431), and all participants provided informed consent. The inclusion criteria were male patients aged 50–80 years, with an American Society of Anesthesiologists physical status < III, scheduled for TURP. The exclusion criteria were patients with anticoagulation or antithrombotic therapy, DIC, procoagulopathy, or hypercoagulable conditions. These exclusion criteria specifically controlled for preoperative factors known to influence platelet function, such as anticoagulant and antithrombotic medications, as well as underlying coagulopathies. All patients were evaluated preoperatively to confirm that none were receiving medications known to significantly affect platelet activity (e.g., aspirin, clopidogrel, and non-steroidal anti-inflammatory drugs) or had comorbidities like severe cardiovascular disease or diabetes mellitus known to substantially alter platelet responsiveness. Preoperative coagulation profiles, including platelet count, prothrombin time (PT), activated partial thromboplastin time (aPTT), and international normalized ratio (INR), were within normal ranges for all included patients.

We collected prostate tissue samples (0.5–1 mg) during TURP and homogenized them in 100 μL of phosphate-buffered saline (PBS) to form prostate tissue homogenates. The resected prostate tissue was collected immediately after TURP and placed in phosphate-buffered saline (PBS) at 4 °C to preserve tissue integrity. To minimize potential contamination from blood, the tissue samples were briefly rinsed three times in PBS before homogenization. This washing step helped remove any loosely adherent blood components while maintaining the biological characteristics of the prostate tissue. Blood samples were collected from the patients (n = 12) after spinal anesthesia induction, with blood drawn from either the great or small saphenous vein to minimize discomfort. Using a two-syringe technique, the first 2 mL of blood was discarded to avoid contamination, and approximately 25 mL of blood was collected. The time from blood collection to the start of experiments (including TURP duration, tissue collection, weighing, and homogenization) was approximately 1–2 h. During this period, blood samples were stored undisturbed to minimize handling-induced platelet activation. All blood samples were anticoagulated with a 1:9 volume of 3.8% sodium citrate solution. From each patient, 10 mL of blood was used for whole-blood experiments to detect platelet–leukocyte aggregation, whereas 15 mL was centrifuged at 200 g for 20 min at 25 °C to obtain platelet-rich plasma.

### 2.3. Study Grouping

The study was divided into four distinct experimental groups, each designed to evaluate different factors influencing platelet activation and platelet–leukocyte aggregation:

1. Group A: The 0.9% saline control group, with no stimulating agents introduced. This group provided baseline measurements for platelet P-selectin expression and platelet–leukocyte aggregation without any external activators.
2. Group B: The thrombin stimulation group. Thrombin was introduced at a final concentration of 0.2 U/mL to assess its role in inducing platelet activation and platelet–leukocyte aggregation. Thrombin is a known platelet activator and was tested to determine its effect in potentially inducing DIC through platelet activation mechanisms.
3. Group C: The PBS control group, with platelets exposed only to PBS. PBS was specifically selected as a control for the prostate homogenate group because the prostate tissues were homogenized in PBS, necessitating an identical buffered environment as a baseline. PBS maintains a stable physiological pH (~7.4), which minimizes pH-induced alterations in platelet function. In contrast, Group A (0.9% saline) served as the control for thrombin stimulation because thrombin was dissolved directly in saline without buffering components, providing an isotonic environment (slightly acidic pH ~5.5–7.0) without additional factors. This design ensures accurate attribution of any observed platelet activation effects to either prostate tissue-specific factors or thrombin itself, without confounding factors related to buffer presence or pH stability.
4. Group D: The prostate tissue homogenate group. Prostate tissue samples (0.5–1 mg) from each patient were collected during TURP and homogenized in PBS (300 µL). The mixture was transferred to centrifuge tubes and homogenized using a homogenizer set at 9000–12,000 rpm for 10 s at 4 °C, with a 60-s pause between each cycle to avoid protein denaturation. This process was repeated until no tissue residue remained. Subsequently, the homogenized prostate tissue was used to investigate its effects on platelet P-selectin expression and platelet–leukocyte aggregation.

**Figure 1:**
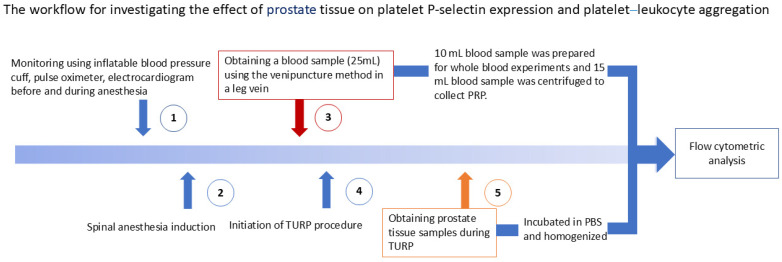
Workflow for investigating the effect of prostate tissue on platelet P-selectin expression and platelet–leukocyte aggregation. After entering the operating room, (**1**) standard monitoring, including non-invasive blood pressure, electrocardiography (lead II), and pulse oximetry, was applied to each patient. (**2**) Spinal anesthesia was induced. (**3**) After anesthesia induction, the blood sample was collected from a lower leg vein by using venipuncture. (**4**) Transurethral resection of the prostate (TURP) was initiated, and (**5**) prostate tissue samples were collected intraoperatively. Both blood and prostate tissue samples were promptly sent to the laboratory for flow cytometric analysis.

Flow cytometry analysis was used to measure platelet P-selectin expression and platelet–leukocyte aggregation, including specific subpopulations: platelet–neutrophil, platelet–monocyte, and platelet–lymphocyte aggregations (Figure 1).

### 2.4. Preparation of Platelet-Rich Plasma and Quantification of P-Selectin Expression in Response to Thrombin and Prostate Tissue Homogenates

**Figure 2:**
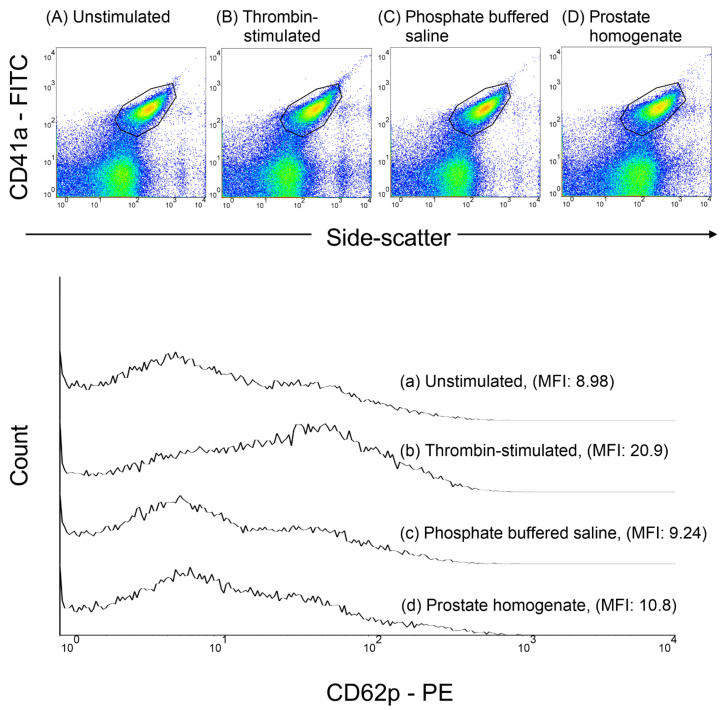
Flow cytometric analysis of platelet P-selectin (CD62P) expression in response to thrombin and prostate tissue homogenates. The upper panel displays dot plots of four experimental conditions: (**A**) unstimulated, (**B**) thrombin-stimulated, (**C**) phosphate-buffered saline (PBS) control, and (**D**) prostate homogenate-treated platelets. The platelets were identified based on CD41a expression (*y*-axis), with side scatter represented on the *x*-axis. The lower panel presents stacked histograms of P-selectin expression for clear and direct comparison between experimental conditions. This histogram arrangement, widely adopted in flow cytometry studies, minimizes overlap and facilitates intuitive visualization of fluorescence intensity shifts across conditions. MFI, mean fluorescence intensity.

Platelet-rich plasma was obtained by centrifuging whole blood at 200 g for 10 min at room temperature. Thrombin or homogenized prostate tissue (0.5–1 mg) was added to the platelet-rich plasma and incubated at 37 °C for 30 min. To assess P-selectin expression, platelet-rich plasma samples were stained with a saturating concentration of anti-CD41a-FITC and anti-CD62P-PE monoclonal antibodies, followed by incubation in the dark at room temperature (24–28 °C) for 20 min. After staining, the samples were fixed with 1% paraformaldehyde at 4 °C for 30 min. Subsequently, flow cytometry was used to analyze P-selectin expression. The platelets were identified based on side-scatter properties and CD41a-FITC fluorescence, and the results are presented as the mean fluorescence intensity (MFI) of CD62P. A minimum of 10,000 platelet events were collected for each sample (Figure 2).

### 2.5. Preparation of Whole Blood and Quantification of Platelet–Leukocyte Aggregations in Response to Thrombin and Prostate Tissue Homogenates

**Figure 3:**
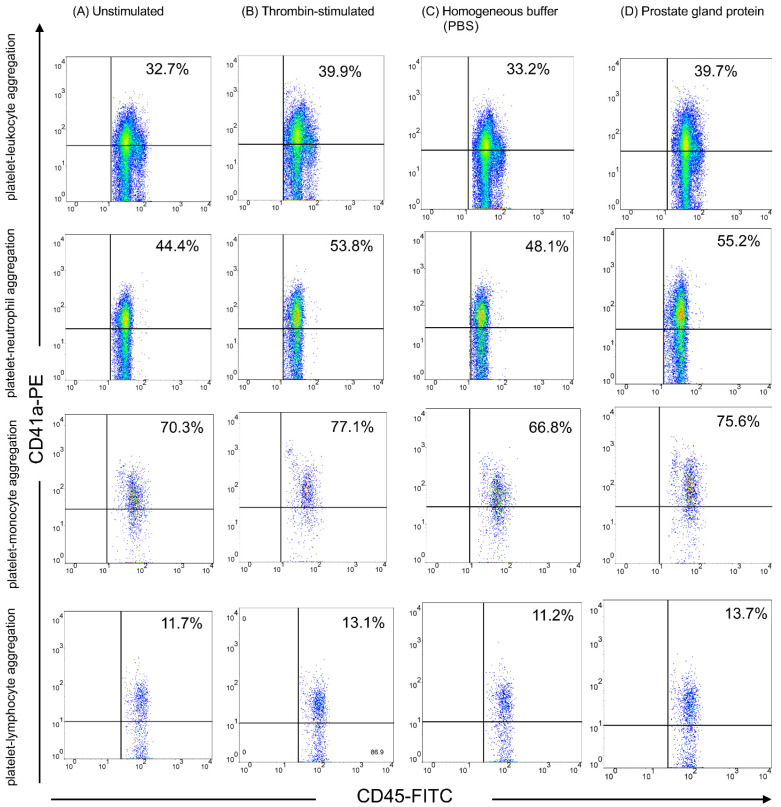
Platelet–leukocyte aggregation and subpopulations in response to prostate tissue homogenate and thrombin stimulation. Flow cytometric analysis of platelet–leukocyte aggregation is presented across four experimental groups: (**A**) unstimulated, (**B**) thrombin-stimulated, (**C**) phosphate-buffered saline control, and (**D**) prostate homogenate-treated platelets. The platelets and leukocytes were identified using CD41a-PE and CD45-FITC, respectively. Aggregation subtypes include platelet–neutrophil, platelet–monocyte, and platelet–lymphocyte conjugates. The figure displays dot plots representing the percentage of each aggregation type in the respective conditions.

Anticoagulated whole blood (10 mL) was preincubated with thrombin or prostate tissue homogenates (0.5–1 mg) for 20 min at room temperature (22–28 °C). For each experiment, 100 µL of platelet-rich plasma (PRP) or 500 µL of whole blood was used. The volume of added reagents was as follows: for the PBS control, 10 µL of PBS was added; for the prostate tissue homogenate condition, 10 µL of homogenized prostate tissue (prepared in PBS at a concentration of 1 mg/100 µL) was added; and for the thrombin-stimulated samples, thrombin was added to achieve a final concentration of 0.2 U/mL in a total volume of 110 µL (for PRP) or 510 µL (for whole blood). After incubation, red blood cells were lysed using FACS™ lysing solution (BD) for 20 min. The samples were centrifuged at 2000 rpm for 6 min, the supernatant was discarded, and the pellet was resuspended in PBS. This washing step was repeated, and the final pellet, containing platelet–leukocyte aggregates, was resuspended in PBS. To detect platelet–leukocyte aggregates, the samples were stained with anti-CD45-FITC (for leukocytes) and anti-CD41a-PE (for platelets) monoclonal antibodies and incubated for 20 min in the dark. Subsequently, the samples were fixed with 1% paraformaldehyde at 25 °C for 30 min and analyzed immediately via flow cytometry. The two-color analysis (CD41a-PE versus CD45-FITC) discriminated platelet-bound from platelet-free leukocytes, and we calculated the percentage of platelet-bound leukocytes in the leukocyte population. Subpopulations of platelet–leukocyte aggregates (platelet–neutrophil, platelet–monocyte, and platelet–lymphocyte aggregates) were further analyzed using forward and side scatter for cell size and cell granularity, respectively (Figure 3). Finally, 10,000 leukocytes were counted in each sample.

### 2.6. Statistical Analysis

The results are presented as the mean ± standard deviation (SD). GraphPad Prism version 9.0 (GraphPad Software, La Jolla, CA, USA) was used for statistical analyses. Normality of the data was assessed using the Shapiro–Wilk test. Because the variables did not follow a normal distribution, non-parametric statistical analyses were employed. Comparisons between two matched groups (group B vs. group A and group D vs. group C) were performed using the paired Mann–Whitney U test (Wilcoxon matched-pairs signed rank test). Statistical significance was considered at *p* < 0.05. All experiments were repeated in triplicate (technical replicates) to ensure reproducibility, and results from the technical replicates were averaged to minimize intra-sample variability and enhance data reliability. The primary outcome measures included platelet P-selectin expression and platelet–leukocyte aggregation, particularly platelet–neutrophil, platelet–monocyte, and platelet–lymphocyte aggregations.

## 3. Results

### 3.1. Demographics of the Study Population

We included 12 patients undergoing TURP. Table 1 shows their demographic characteristics, including age, body mass index, and other relevant parameters.

### 3.2. P-Selectin Expression in Response to Prostate Tissue Homogenates and Thrombin Stimulation

**Figure 4:**
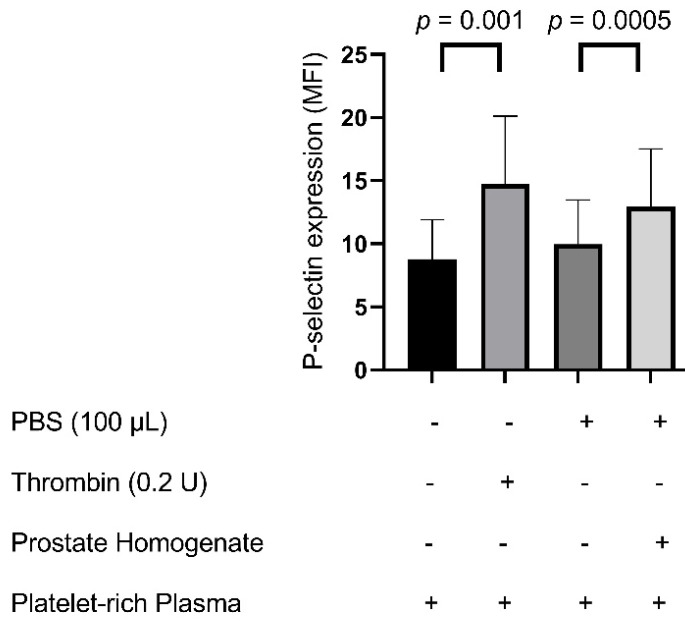
P-selectin expression in response to prostate tissue homogenates and thrombin stimulation. Flow cytometry was used to measure the mean fluorescence intensity (MFI) of P-selectin (CD62P) in platelet-rich plasma samples. Four experimental conditions were assessed: unstimulated platelet-rich plasma (black bars), thrombin-stimulated platelet-rich plasma (light gray bars), phosphate-buffered saline (PBS) control (dark gray bars), and platelet-rich plasma treated with prostate homogenates (white bars). The “+” and “–” symbols in the table below the graphs indicate the presence (+) or absence (–) of each component used in the experimental condition: phosphate-buffered saline (PBS, 100 µL), thrombin (0.2 U), prostate homogenate, and platelet-rich plasma. *p*-values indicate statistical significance between groups. Data are presented as the mean ± SD. Statistical comparisons were performed using paired non-parametric tests.

Flow cytometric analysis revealed that P-selectin (CD62P) expression significantly increased following exposure to both prostate tissue homogenates and thrombin stimulation compared with the PBS control (*p* < 0.05) (Figure 4). Specifically, significant differences were observed in group B vs. group A (14.76 ± 5.35 vs. 8.78 ± 3.13, *p* = 0.001) and group D vs. group C (12.93 ± 4.58 vs. 9.97 ± 3.51, *p* = 0.0005).

### 3.3. Platelet–Leukocyte Aggregation and Subpopulations

**Figure 5:**
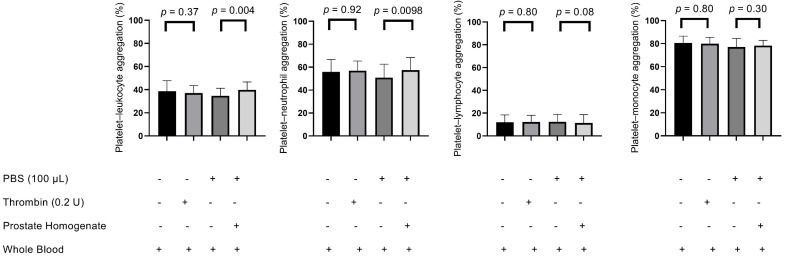
Effect of prostate tissue on platelet–leukocyte aggregation subpopulations in response to thrombin stimulation. Flow cytometry was used to assess platelet–leukocyte aggregation (including platelet–neutrophil, platelet–monocyte, and platelet–lymphocyte aggregations). The experimental groups include unstimulated whole blood (black bars), thrombin-stimulated whole blood (light gray bars), phosphate-buffered saline (PBS) control (dark gray bars), and whole blood treated with prostate homogenates (white bars). The “+” and “–” symbols in the table below the graphs indicate the presence (+) or absence (–) of each component used in the experimental condition: phosphate-buffered saline (PBS, 100 µL), thrombin (0.2 U), prostate homogenate, and whole blood. The data represent the percentage of each aggregation subpopulation. Data are presented as the mean ± SD. Statistical comparisons were performed using paired non-parametric tests.

The percentage of platelet–leukocyte aggregations increased significantly following treatment with prostate tissue homogenates compared with the PBS control (group D vs. group C: 39.74 ± 6.92 vs. 34.71 ± 6.52, *p* = 0.004) (Figure 5). However, no significant difference was observed in the thrombin-stimulated group compared with its saline control (group B vs. group A: 38.64 ± 9.04 vs. 37.16 ± 6.47, *p* = 0.37). Moreover, platelet–neutrophil aggregation was particularly elevated in the prostate homogenate-treated groups (group D vs. group C: 57.41 ± 10.98 vs. 50.82 ± 11.69, *p* = 0.0098), whereas no significant difference was found between the thrombin-stimulated group and its control (group B vs. group A: 56.94 ± 8.47 vs. 55.86 ± 10.75, *p* = 0.92). Platelet–lymphocyte aggregation showed no significant differences in either the thrombin-stimulated (group B vs. group A: 12.17 ± 6.00 vs. 11.92 ± 6.54, *p* = 0.80) or prostate tissue homogenate-treated samples (group D vs. group C: 11.28 ± 7.41 vs. 12.39 ± 6.63, *p* = 0.08). Similarly, platelet–monocyte aggregation revealed no significant differences in thrombin-stimulated (group B vs. group A: 79.92 ± 5.66 vs. 80.62 ± 5.88, *p* = 0.80) or prostate tissue homogenate-treated groups (group D vs. group C: 78.33 ± 4.57 vs. 77.05 ± 7.48, *p* = 0.30). (*p* = 0.0098).

## 4. Discussion

Prostate tissue homogenates can significantly induce platelet P-selectin expression and platelet–neutrophil aggregation, similar to the findings observed with thrombin stimulation. P-selectin was chosen as the primary marker of platelet activation due to its well-established role as an early and specific indicator of platelet degranulation. Upon platelet activation, P-selectin rapidly translocates from α-granules to the platelet surface, mediating critical interactions with leukocytes and endothelial cells. Elevated P-selectin expression is closely associated with inflammatory and thrombotic states, including disseminated intravascular coagulation (DIC), sepsis, and cardiovascular diseases, making it a clinically meaningful biomarker of platelet-mediated thrombo-inflammatory responses [7,13,14,15]. Additionally, the measurement of platelet P-selectin expression via flow cytometry is a reliable, sensitive, and widely validated method, enabling robust and reproducible comparisons with the existing literature. Although nonspecific platelet activation may theoretically occur with any tissue homogenate, we utilized phosphate-buffered saline (PBS) and saline as controls to address this concern. The significantly greater platelet activation seen in the prostate tissue homogenate group, compared to these controls, suggests that the observed response was not merely due to mechanical manipulation or nonspecific foreign material exposure. Moreover, prostate tissue homogenates induced platelet activation and aggregation to an extent comparable to that induced by thrombin, suggesting the presence of prostate-specific procoagulant or inflammatory factors rather than a generic response to tissue homogenization alone. However, total platelet–leukocyte aggregation, including platelet–monocyte and platelet–lymphocyte aggregations, did not show a significant response to prostate tissue exposure, indicating that prostate tissue, particularly during TURP, may contribute to DIC through mechanisms involving thrombin generation. Increased platelet P-selectin expression and platelet–neutrophil aggregation in response to prostate tissue homogenates indicate that DIC may develop due to platelet activation [11,13,15,16], immune responses, and inflammation (mediated by cytokines and inflammatory cells) [17,18,19]. Additionally, thrombin and its influence on platelet–leukocyte interactions through enhanced P-selectin expression aligns with our study results [7,8,9,11,12,13]. These mechanisms, along with endothelial injury and tissue factor release, complement each other in contributing to DIC development [7,8,9,11,12,13].

Our findings align with earlier research highlighting coagulation disturbances following TURP. Prior clinical studies have reported that TURP surgery may enhance both coagulation and fibrinolytic activity, thereby increasing the risk of bleeding [20]. Additionally, intraoperative absorption of irrigation fluids during TURP has been shown to disrupt blood coagulation cascades either through dilutional effects or interference with coagulation factor activity [6]. These previous clinical studies support our hypothesis that prostate tissue itself may have the capacity to induce platelet activation and aggregate specific procoagulant factors, potentially contributing to clinically relevant coagulopathy following surgery. During TURP, prostate tissues come into direct contact with blood, introducing factors, such as thrombin, into circulation, exacerbating platelet activation. Thrombin is a key mediator of platelet P-selectin expression and has been implicated in inflammatory responses linked to DIC [7,8,9,11,12,13,14]. Our data support this mechanism, showing significant increases in platelet–neutrophil aggregation following exposure to both thrombin and prostate tissue homogenates, which is consistent with prior research, which highlights the central role of thrombin in amplifying platelet–leukocyte interactions during systemic inflammation [15,18,21]. Although both thrombin and prostate tissue homogenates elicit similar platelet responses, the homogenates may contain additional prostate-specific factors that modulate platelet activity

Thrombin plays a major role in developing DIC. By using thrombin rather than thrombin receptor-activating peptide (TRAP) in our study, we aimed to closely mimic physiological conditions encountered during TURP. In future research, we could consider using TRAP or specific coagulation inhibitors to further elucidate these mechanisms and to differentiate platelet activation from coagulation-driven effects. Nevertheless, other inflammatory cytokines or coagulation activators could also provide valuable insights into the complex interplay between inflammation and coagulation. Our findings indicate that thrombin-induced increase in platelet P-selectin expression may ignite the platelet–neutrophil aggregation process. TURP in patients with BPH has been associated with thrombin generation [20]. Thrombin increases platelet P-selectin expression, enhancing platelet–leukocyte aggregation [7]. Consequently, TURP can promote P-selectin expression and platelet–neutrophil aggregation through thrombin activation. Elevated platelet P-selectin levels may drive the increased platelet–neutrophil aggregation [22]. The interaction between thrombin, P-selectin, and platelet–neutrophil aggregation could lead to DIC [7,13,20,21,22]. Our findings further suggest that platelet–neutrophil aggregation is more sensitive to thrombin than platelet–monocyte and platelet–lymphocyte aggregations. Therefore, both prostate tissue homogenates and thrombin are causally related to inducing platelet P-selectin activation and platelet–neutrophil aggregation, potentially leading to DIC. In DIC, ongoing coagulation stimulation, mediated by continuous exposure to tissue factor, thrombin, or other procoagulant substances, places patients at risk of thrombosis. Moreover, DIC can develop when blood is exposed to large amounts of tissue factor or other procoagulants over a short period, resulting in substantial thrombin generation [8]. Hence, prostate tissue exposure may induce P-selectin expression through the tissue factor pathway [8,23].

Despite the robust response in platelet–neutrophil aggregation, indicating that prostate tissue and thrombin selectively promote platelet–neutrophil interactions, potentially through P-selectin glycoprotein ligand-1 (PSGL-1)-mediated pathways, the specificity of this interaction suggests a key mechanism by which DIC develops in response to tissue injury and inflammation during TURP, where platelet–neutrophil aggregates play a crucial role in the procoagulant and inflammatory responses [22]. Our results showed that prostate tissue homogenates can induce platelet P-selectin activation and platelet–neutrophil aggregation, which may contribute to DIC development. Increased platelet P-selectin levels can lead to further platelet–neutrophil aggregation [22], which supports the idea that prostate tissue can drive mechanisms that link hemostasis and inflammatory processes [17].

During septicemia, interactions between platelets and neutrophils initiate a detrimental feedback loop that sustains neutrophil extracellular trap formation, DIC, and inflammation [23]. Platelet–leukocyte aggregation mainly occurs through the binding of P-selectin on platelets to PSGL-1 expressed on leukocytes [22]. Thus, the increased platelet–leukocyte aggregation is not solely due to increased platelet P-selectin levels but also to leukocyte activation [22]. Although platelet–leukocyte aggregation depends on leukocyte activation, leukocyte activation was not evaluated in this study. However, platelet–monocyte and platelet–lymphocyte aggregations did not show significant differences in response to prostate tissue homogenates (Figure 5). Therefore, platelet–neutrophil aggregation may be more sensitive than platelet–monocyte and platelet–lymphocyte aggregations in response to prostate tissue homogenates and can contribute to DIC.

In addition to direct platelet activation by prostate tissue and thrombin, transurethral resection of the prostate (TURP) is well documented to elicit a systemic inflammatory response, often characterized by elevated cytokine levels and acute-phase reactants. This proinflammatory milieu may further enhance platelet activation through mechanisms such as endothelial cell activation, upregulation of adhesion molecules, and neutrophil–platelet crosstalk. Although our current in vitro model did not assess systemic inflammatory markers, it is plausible that inflammation and platelet activation act synergistically during TURP, thereby increasing the risk of thrombotic complications, including disseminated intravascular coagulation (DIC). Future studies incorporating measurements of circulating inflammatory biomarkers would be valuable in elucidating the interplay between inflammation and platelet–leukocyte interactions in this clinical context.

Another possible indirect mechanism during TURP that may induce DIC is the interaction of P-selectin and platelet–neutrophil aggregation with the vessel endothelium, which may contribute to DIC [8,9,13,15,16,17]. The prostate contains vessel endothelia, and endothelial perturbation is a crucial factor for most patients with DIC [8,9]. During TURP, the prostate tissue is removed using a resectoscope inserted through the urethra, leading to vessel endothelium injury in the transition zone of the prostate, resulting in the release of procoagulant substances, loss of endothelial antithrombotic properties [8], and promotion of platelet adherence and activation [8], including our findings of increased platelet P-selectin expression [13] and platelet–neutrophil aggregation [15,16,17]. Moreover, endothelial perturbation, along with inflammatory cell activation and inflammatory mediator release, plays a key role in this mechanism [9]. Once endothelial integrity is compromised, mononuclear cells are activated by cytokines and hormonal signals, leading to the upregulation of additional cytokines, surface receptors, procoagulant proteins, and platelets. Consequently, the endothelium shifts from an anticoagulant to a procoagulant surface, and fibrinolysis is inhibited [9]. Thus, this inflammatory cascade can result in microvascular thrombosis, multiorgan dysfunction, and eventually multiorgan failure [9]. Therefore, TURP may increase P-selectin expression and enhance platelet–neutrophil aggregation as a result of injury to the prostate endothelium. Second, the interaction of injured endothelial cells, tissue factor, and P-selectin may contribute to DIC [8,23]. Endothelial cells can release tissue factor, which plays a central role in initiating inflammation-induced coagulation in DIC [8]. DIC is commonly triggered by the exposure of blood to tissue factors, leading to thrombin generation. Third, sepsis is a well-documented complication of TURP [4,24]. Bacteria entering the circulation through the prostatic venous sinuses, together with the use of high-pressure irrigation, can lead to sepsis and subsequently DIC [5]. In addition, the tight junctions of the prostate may be compromised, increasing bacterial permeability in BPH [25]. Sepsis can elevate platelet P-selectin expression [18], and P-selectin is a predictive marker for sepsis outcomes, including the length of stay and 30-day survival [18]. Sepsis commonly induces platelet activation and consumption, leading to thrombocytopenia and DIC [13]. Bacteremia during TURP further contributes to DIC and coagulopathy [24,26]. Considering the infectious risks associated with TURP and its role in increasing platelet P-selectin expression [18], these factors could collectively contribute to DIC development.

Notably, baseline platelet–leukocyte aggregation levels were relatively high in unstimulated samples. This may reflect inherent platelet reactivity or sample handling effects, despite our careful procedures. Mild platelet activation commonly occurs during ex vivo handling, centrifugation, and sample transfer. Additionally, citrate anticoagulation, though effective in preventing coagulation, may not completely inhibit platelet activation during handling, thus elevating baseline aggregation. Some TURP patients may also have a pre-existing inflammatory condition related to BPH or other underlying diseases, contributing to elevated baseline platelet–leukocyte aggregation. Importantly, our results demonstrated clear and significant increases in platelet–neutrophil aggregation in response to both thrombin and prostate tissue homogenates, emphasizing the additive effects beyond these baseline conditions. Future experiments could benefit from employing platelet activation inhibitors, such as anti-P-selectin antibodies, to distinguish spontaneous from stimulus-induced aggregation.

Our findings have clinical implications, particularly in the prevention and management of TURP-related disseminated intravascular coagulation (DIC). Given the strong association between P-selectin expression, platelet–neutrophil aggregation, and thrombin activation observed in our study, these pathways may serve as potential therapeutic targets for mitigating DIC risk in TURP patients. This suggests that perioperative strategies focusing on platelet function modulation, such as targeting P-selectin-mediated interactions or controlling thrombin activation, could help reduce thrombotic complications in high-risk patients undergoing TURP. Further research should aim to identify the specific molecular mediators within prostate tissue that amplify platelet activation and thrombin effects, potentially leading to more precise therapeutic interventions.

Our study has limitations. First, we did not investigate the specific molecular mediators in the prostate tissue that may contribute to the increased platelet P-selectin expression and platelet–neutrophil aggregation. Identifying these mediators would be crucial in understanding the mechanisms behind these findings. Second, during TURP, the prostate tissue or related factors may likely enter the circulation and interact with blood cells and other components. However, we did not quantify the circulating concentration of prostate tissues during or after TURP, which could have provided more insight into the systemic effects of the tissue. Third, although we observed an increase in platelet–neutrophil aggregation, we were unable to assess the neutrophil activation level. While routine preoperative laboratory tests (e.g., neutrophil, platelet, and erythrocyte counts) were available, they do not directly reflect neutrophil activation status. Increased neutrophil activation remains a possibility and could also contribute to DIC development [11,15,16]. Additionally, our study was constrained by a relatively small sample size (n = 12), which may limit the generalizability and reproducibility of our findings. Furthermore, our results are based on an in vitro experimental approach without direct in vivo validation; therefore, the extent to which these platelet activation mechanisms translate into clinical scenarios remains to be established. Finally, due to the study’s design, we were unable to assess long-term patient outcomes, such as persistent coagulation disturbances or thrombotic events, following TURP. Future studies incorporating larger patient cohorts, in vivo validation, and longitudinal outcome assessments are essential to comprehensively evaluate the clinical implications of prostate tissue-induced platelet activation.

## 5. Conclusions

Our study shows that the prostate tissue, in TURP, can significantly induce platelet P-selectin expression and platelet–neutrophil aggregation, indicating that it may contribute to DIC by promoting platelet activation and interaction with leukocytes, particularly neutrophils. Thrombin plays a central role in this process, and our data indicate that both thrombin and prostate tissues can induce similar levels of platelet–neutrophil aggregation. The selective increase in platelet–neutrophil aggregation, without a corresponding increase in platelet–monocyte or platelet–lymphocyte aggregation, highlights the unique inflammatory and prothrombotic response that the prostate tissue elicits during TURP. This response may be a key factor in the development of DIC. Thus, targeting platelet–neutrophil aggregation and P-selectin expression may offer novel therapeutic approaches to mitigate the risk of DIC in these patients. Our study underscores the importance of understanding the coagulation and inflammatory pathways activated by prostate tissues during TURP, with potential clinical implications for preventing serious thrombotic complications, such as DIC. Further studies are required to elucidate the molecular pathways involved and explore potential interventions that could prevent or manage TURP-related DIC.

## Figures and Tables

**Table 1:** Demographic characteristics of patients for detecting platelet–leukocyte aggregation and P-selectin expression in response to in vitro prostate tissue protein treatment.

	Value
No. of cases	12
Age (years)	64.00 ± 6.78
Height (cm)	167.00 ± 6.52
Weight (kg)	71.83 ± 7.86

Data are presented as the mean ± SD.

## Data Availability

The data presented in this study are available from the corresponding author upon request.
